# Bridging academia and industry: advancing systems biology and QSP education through AstraZeneca’s collaborative partnerships

**DOI:** 10.3389/fsysb.2025.1627214

**Published:** 2025-09-12

**Authors:** Cesar Pichardo-Almarza, Holly Kimko

**Affiliations:** ^1^ Systems Medicine, Clinical Pharmacology and Safety Sciences, AstraZeneca, Cambridge, United Kingdom; ^2^ Systems Medicine, Clinical Pharmacology and Safety Sciences, AstraZeneca, Gaithersburg, MD, United States

**Keywords:** quantitative systems pharmacology (QSP), systems biology, industry academia collaboration, MIDD (model-informed drug development), multidiscplinary, education and training

## Abstract

Collaborations between industry leaders and academia are crucial for advancing systems biology education and training. This article explores opportunities for partnerships to enhance the educational landscape and develop a workforce skilled in systems modelling, particularly for quantitative systems pharmacology (QSP) in drug development. Companies with a strong focus on innovation frequently explore collaborative ventures involving joint research, co-designed curricula, and specialized training programs. These partnerships provide students and researchers with insights into real-world applications of systems biology and QSP. We explicitly review success criteria for collaboration at MSc and PhD levels, discuss earlier pipeline considerations, and carefully balance the roles, incentives, and challenges for both academia and industry in collaborative ventures. Challenges in aligning academic and industry objectives exist, including resource allocation and intellectual property concerns. However, the importance of training skilled systems biologists for advancing drug discovery and development outweighs these challenges. The article concludes by highlighting successful industry-academia partnerships and offering recommendations for optimizing collaborations to meet the evolving needs of systems biology education and drive innovation in pharmaceutical research.

## 1 Introduction

Systems Biology (SB) and Quantitative Systems Pharmacology (QSP) have emerged as critical disciplines in modern drug development, offering a transformative approach to understanding complex biological systems (Kitano, 2002). SB constructs comprehensive models of biological processes by incorporating data from multiple levels of data, such as molecular, cellular, organ, and organism levels (Hood et al., 2004; Joyce and Palsson, 2006). This holistic view enables researchers to gain a deeper understanding of disease mechanisms and predict how drugs will interact with the human body (Butcher et al., 2004). QSP leverages these models to simulate drug behaviours, predict patient responses, and optimize drug development strategies (Vicini and van der Graaf, 2013). By incorporating QSP into the drug discovery process, pharmaceutical companies can make more informed decisions, reduce development costs, and ultimately bring safer and more effective therapies to patients (Gadkar et al., 2016; Musante et al., 2017).

Given this landscape, our discussion primarily focuses on MSc and PhD-level training, which are essential for developing the specialist expertise needed for research and technical roles in SB and QSP. Although some undergraduate (BSc) programmes have started to include relevant modules, there remains significant potential to further expand and unify specialist training across educational levels and institutions.

Several universities have already developed MSc programmes or dedicated electives relevant to SB and QSP, including:

• University of Manchester: MSc Bioinformatics and Systems Biology (The University of Manchester, 2025a)
• Imperial College: MSc in Systems and Synthetic Biology (Imperial College London, 2025)
• Maastricht University: MSc Systems Biology and Bioinformatics (Maastricht University, 2025)
• University of Amsterdam: MSc Bioinformatics and Systems Biology (University of Amsterdam, 2025)
• Wageningen University and Research: Master’s Bioinformatics (Wageningen University and Research, 2025)
• University of Delaware: MSc in Quantitative Systems Pharmacology (University of Delaware, 2025)
• University at Buffalo: MS in Pharmacometrics and Personalised Pharmacotherapy, including an elective course on QSP (University at Buffalo, 2025)
• University of Florida: Online Graduate Programme in Pharmaceutics, including a course on QSP (University of Florida, 2025)

These programmes provide valuable frameworks for specialist training and serve as important models as the field continues to evolve and expand.

The increasing complexity of drug development necessitates a highly skilled workforce with expertise in SB and QSP. These individuals should possess a unique blend of biological, mathematical, and computational skills, enabling them to develop complex models, interpret data, and translate scientific knowledge into actionable insights. The demand for such skilled professionals is growing rapidly as the pharmaceutical industry increasingly embraces model-based drug development approaches. While these emerging specialist programmes represent significant progress, broader integration of SB and QSP principles across traditional academic curricula would help ensure that more students develop the foundational skills needed to thrive in this dynamic field (Beattie et al., 2024; Gadkar et al., 2016).

To bridge this gap and cultivate a robust workforce, fostering strong collaborations between industry leaders and academic institutions is crucial. These partnerships can provide invaluable opportunities for students and researchers to gain practical experience, access cutting-edge technologies, and contribute to real-world research challenges. By working together, academia and industry can develop innovative educational programs that integrate theoretical knowledge with practical applications, equipping the next-generation of scientists with the skills and expertise needed to advance the field of systems biology and QSP. We envision success in these collaborations to be demonstrated by increases in joint enrolments, diversity of student backgrounds, new joint publications and outputs, and enhanced student transitions to industry or postdoctoral roles (Nijsen et al., 2018). Long-term impact could be measured by tracking graduate placements, feedback, and evidence of influential advances made by alumni who received collaborative, practice-exposed training.

This paper explores how joint initiatives between industry and academia can simultaneously enrich the educational landscape, create a systems modelling-ready workforce, and drive the faster development of novel therapies, benefiting both sectors. Importantly, we address the need for greater specificity in examples of successful curricular models and teaching strategies, as identified in recent reviews (Androulakis, 2022), ensuring that our discussion extends beyond generalities and offers actionable insights for curriculum designers and instructors.

## 2 Enhancing education through industry-academia partnerships

### 2.1 Models for collaboration

**FIGURE 1:**
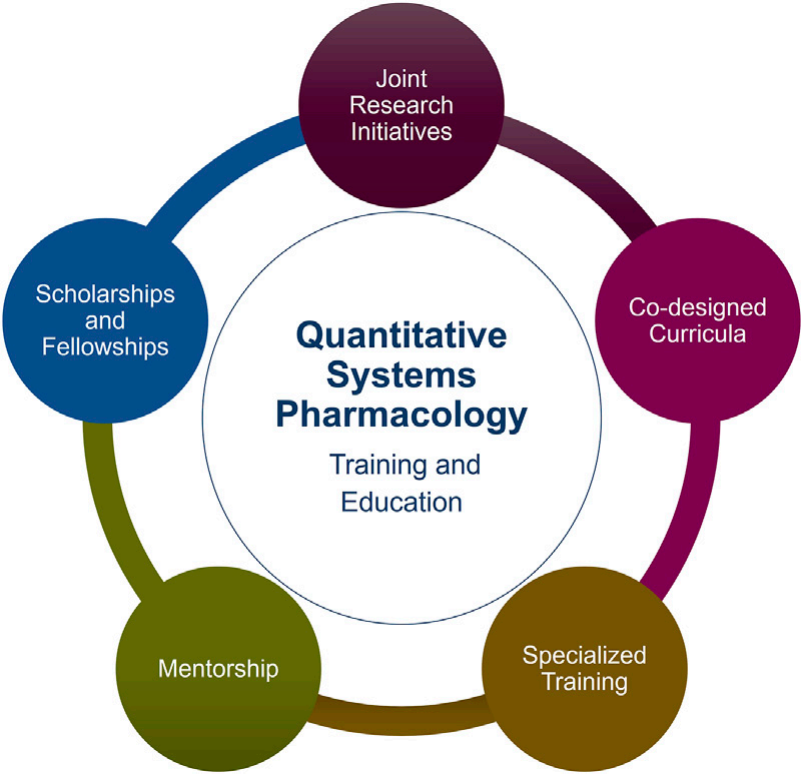
Potential collaborations between industry and Academia for QSP training and education

The most transformative partnerships typically combine several elements (Figure 1), which we now discuss using the following structure: each collaborative avenue is examined in terms of its current challenges/barriers, a practical case study or exemplar, and recommendations for best practice.

#### 2.1.1 Co-designed academic curricula

##### 2.1.1.1 Current challenges

Integrating relevant SB and QSP content into the formal curriculum at the MSc and PhD levels can be hampered by limited faculty with relevant applied experience, inflexibility of traditional university structures, and resource constraints. Student awareness of real-world applications remains uneven. While post-graduate offerings are growing, there is an acknowledged gap at the undergraduate level, where foundational knowledge in computational biology and pharmacology must be established. Existing undergraduate-level courses are still few, and their construction can be challenging due to a lack of established templates or exemplars.

Integrating SB and QSP into formal MSc and PhD curricula faces significant hurdles. These include a scarcity of faculty possessing practical, applied experience in these specialized areas; the difficulty to adapt to rapidly evolving scientific disciplines in traditional university structures; and pervasive resource constraints that limit the development of new programs and courses. Furthermore, a foundational challenge lies in student unfamiliarity with SB and QSP. Many students enter higher education without prior exposure to these fields at the undergraduate level, which subsequently limits their consideration of SB and QSP as viable paths for advanced study. Therefore, to cultivate a robust pipeline of talent and ensure future scientific literacy, it is imperative to introduce the fundamental principles of SB and QSP during undergraduate programs, thereby building a foundational understanding that encourages deeper engagement at the graduate level.

##### 2.1.1.2 Case study/exemplar

At the University of Manchester, the MSc in Model-based Drug Development (The University of Manchester, 2025b) integrates real-world case studies informed by current industry practice and research. The programme combines theoretical teaching with hands-on modelling and data analysis projects, and is designed and delivered with strong input from industry experts, including guest lectures and contributions from practising scientists in the field. In Dutch MSc Systems Biology programmes, such as those at Maastricht University and Wageningen University, industrial partners play an active role by providing real-life case studies, co-supervising research and group projects, and offering practical experience (Maastricht University, 2025; Wageningen University and Research, 2025).

Notably, recent work by Androulakis (2022)presents a detailed framework for constructing undergraduate courses that bridge computational systems biology and quantitative pharmacology. This model draws on specific learning modules, including mathematical modelling, numerical simulation, pharmacokinetics and pharmacodynamics, and highlights pedagogical approaches such as interdisciplinary project work and authentic, clinically-relevant problem scenarios. Androulakis provide actionable, modular syllabi and assessment strategies that can be directly adopted or adapted by other institutions looking to launch or refine similar offerings. Importantly, the curriculum addresses both “why” (the rationale and employability benefits), “who” (target learner backgrounds), and “what” (specific topics, mathematical tools, and biological context) questions, providing a concrete roadmap for faculty and administrators seeking to initiate undergraduate coursework in this area. By referencing and building upon such curriculum-focused scholarship, collaborative efforts can move beyond broad recommendations and implement proven educational models with a record of successful student outcomes.

##### 2.1.1.3 Recommendation

Embedding up-to-date industrial case studies and industry co-teaching into MSc/PhD curricula builds stronger practical understanding and exposes students to evolving needs. Partnerships that co-design assessment and accreditation (with employability as an explicit goal) are more sustainable and impactful. Challenge events such as iGEM (2025) provide cross-institutional opportunities for teams to tackle real-world problems, often with both industry and academic mentors, further exemplifying the value of shared educational design.

#### 2.1.2 Specialized training and experiential programmes

##### 2.1.2.1 Current challenges

While workshops and internships are valuable, opportunities are limited by funding, access to industry, and awareness among students. Ensuring these experiences have academic recognition and don’t duplicate existing advisory services or become “tick-the-box” offerings is vital.

##### 2.1.2.2 Case study/exemplar

AstraZeneca hosts competitive summer internships (open to MSc and PhD students), where students work alongside multi-disciplinary project teams. Outcomes include exposure to high-impact SB/QSP problems, chance to publish jointly, and the development of networks that often lead to post-graduation employment or further fellowships. The company also supports long-term (year-long) “sandwich” placements for undergraduates and postgraduates.

##### 2.1.2.3 Recommendation

Expand internship and placement schemes through central, transparent processes so that students from a range of institutions (including non-partner universities) can access them. Both short, challenge-based events (“datathons”, “hackathons”) and longer embedded internships are crucial. Academic credit can and should be given for these structured industrial experiences wherever possible.

#### 2.1.3 Mentorship and career development

##### 2.1.3.1 Current challenges

Mentorship by industry experts offers invaluable exposure to new perspectives but may conflict with, or duplicate, roles of existing academic advisors. Coordination and role clarity are essential to avoid redundancy and maximize student benefit. There can be cultural barriers to integrating such mentorship, especially where universities value academic autonomy and independence.

##### 2.1.3.2 Case study/exemplar

Industrial CASE PhDs in the United Kingdom (UKRI, 2025), pair each student with both an academic supervisor and an industry-based mentor, who together co-guide the project across the student’s tenure. Evaluation of these schemes shows higher employability and a broader skillset among alumni (Nijsen et al., 2018).

##### 2.1.3.3 Recommendation

Define clear roles for industry mentors—complementary to, but not replacing, university career advisors or supervisors. Effective schemes have regular triage meetings between all parties (student/university advisor/industry mentor) and shared reporting on student goals and outcomes. Industry-led career development events targeting pre-university (e.g., through AstraZeneca STEM outreach), undergraduate, and postgraduate students ensure the SB and QSP career pipeline does not narrow too early for lack of information or encouragement.

#### 2.1.4 Industry support for academic training: scholarships and fellowships

##### 2.1.4.1 Current challenges

Financial constraints limit access to advanced training for students from underrepresented groups or international backgrounds. Balancing broad sectoral impact with targeted industrial training needs is a further challenge.

##### 2.1.4.2 Case study/exemplar

AstraZeneca and related sectors offer competitive fellowships/scholarships for MSc/PhD students, often including funding for conference travel or international placements. In the United Kingdom, in particular for PhD students, these awards are administered in parallel with, or sometimes through, professional societies and research councils, with examples including support for Centres for Doctoral Training (UKRI, 2023) and industrial sponsorship of PhD programmes (University of Cambridge, 2025) —maximizing expertise and fairness.

##### 2.1.4.3 Recommendation

Expand funding to support broader, inclusive access to SB/QSP research training. Industry should co-fund competitive scholarships but also support travel and innovation awards for students at every level (including high school outreach), as part of a systemic effort to widen participation.

### 2.2 Broader infrastructure: academic societies and funding bodies

Scientific societies and funding bodies increasingly act as catalysts for industry-academia collaboration. For example, several scientific societies like International Society of Pharmacometrics (ISoP) and American Society of Clinical Pharmacology & Therapeutics (ASCPT) include industrial voices within advisory and working groups, shaping training, research prioritization, and public engagement agendas. Similarly, funding bodies such as the EU’s Innovative Medicines Initiative (IMI) require industry-academia consortia for eligibility—driving systematically collaborative modes of research and education.

We recommend all academic SB/QSP groups maintain advisory boards with practitioner (industry) representation, ensuring curriculum and career preparation evolve with the field’s needs. Funding bodies should continue to suggest cross-sectoral partnerships for major SB/QSP training grants.

## 3 Balancing goals and expectations: aligning academia and industry

### 3.1 Current challenges

There exists a perception—and sometimes a reality—of cultural and operational differences between academia and industry, especially in research planning and time horizons. While industry projects may operate under commercial timelines and deliverables, academic research is also constrained by time (e.g., PhD and MSc thesis deadlines) and increasingly by funding cycles. Academic research’s independence, particularly regarding topic selection and dissemination, is often cited as a key benefit; equally, industry offers practical problem-solving skills and stringent project management, which are transferrable and highly valued in modern research careers.

As highlighted by the United Kingdom Research and Innovation (UKRI) students in industrial CASE PhDs (UKRI, 2025) gain the benefits of both environments: exposure to applied problem-solving and data resources from industry, alongside academic freedom and methodological rigor. Academic projects without industrial input can sometimes lack immediate application, but are well-positioned for foundational innovation for future career in academia or industry. Conversely, industry projects—while impactful—may be deprioritised if commercial outcomes become uncertain. Therefore, the most effective collaborations should intentionally set and review shared aims, and flexibly adjust communication and deliverables as needs evolve (EFPIA MID3 Workgroup et al., 2016).

## 4 Successful case studies: partnerships, consortia and educational programs

AstraZeneca actively fosters collaborations with academic institutions and participates in industry-academia consortia, leading to notable progress in QSP, systems biology, and Model-Informed Drug Discovery and Development (MID3).

AstraZeneca’s engagement with academia extends beyond formal research projects to encompass involvement in industry-academia consortia, professional societies, and various educational and career development initiatives, including lectures in universities. Additionally, AstraZeneca supports academic sabbaticals and visiting scholar positions, further promoting knowledge transfer and nurturing enduring relationships between industry and academia. These multifaceted engagement programs, consortium participations, and involvement in professional societies collectively contribute to the development of future pharmaceutical researchers and strengthen connections among AstraZeneca, academic institutions, and regulatory bodies, creating a vibrant ecosystem for innovation in drug research and development, particularly within QSP and related modeling and simulation domains. The subsequent section provides brief examples of these collaborations in action.

In recent years, AstraZeneca has established collaborations with diverse academic groups across various therapeutic areas and modelling approaches to develop projects aimed at advancing the evaluation of drug efficacy (QSP) and toxicity (QST), fostering innovation and knowledge exchange between industry and academia. While not exhaustive, some examples include cardiovascular modelling to study cardiomyopathy and contractility (University of Oxford, United Kingdom), microcirculation in solid tumours (University of Ghent, Belgium), Antibody Drug Conjugate safety modelling (University at Buffalo, US), modelling oncology combinations (University of Virginia), gastrointestinal organoid modelling (University of Nottingham) and renal modelling (University of Georgia, US). These collaborations involve PhD students and postdoctoral researchers, providing valuable training opportunities and fostering the next-generation of quantitative systems pharmacologists.

AstraZeneca’s commitment to academic partnerships extends far beyond this list. The company actively engages in a variety of collaborative efforts, including participation in industry-academia consortia, involvement in professional societies, and support for educational programs. The following sections explore AstraZeneca’s involvement in key consortia, its participation in professional societies, and its support for educational programs.

### 4.1 Industry-academia consortia and professional society participation

1. Simcyp Consortium: AstraZeneca is a long-standing member, focusing on developing and improving physiologically-based pharmacokinetic (PBPK) modeling approaches (Jamei et al., 2009; Rostami-Hodjegan and Tucker, 2007).
2. TransQST Consortium: As part of this Innovative Medicines Initiative (IMI) project, AstraZeneca collaborates on developing novel computational approaches for translational safety assessment (Beattie et al., 2024; Ferreira et al., 2020; Gall et al., 2023).
3. IQ Consortium: AstraZeneca participates in working groups focused on various aspects of drug development, including modeling and simulation (Nijsen et al., 2018).
4. Center for Protein Therapeutics (University at Buffalo): AstraZeneca is a member of this consortium, which advances quantitative pharmacology methods, including pharmacometrics, PBPK modeling, and systems pharmacology (University at Buffalo, 2024).
5. IMI-EFPIA DDMoRe Consortium: AstraZeneca contributed to this initiative aimed at developing a public drug and disease model library and standards for model exchange (Harnisch et al., 2013).
6. EFPIA MID3 Workgroup: While not a formal consortium, AstraZeneca participates in this workgroup focusing on Model-Informed Drug Discovery and Development (MID3) practices (Workgroup et al., 2016).
7. Critical Path Institute (C-Path) Consortia: AstraZeneca is involved in several C-Path consortia, including the Quantitative Systems Pharmacology Consortium and the Translational Therapeutics Accelerator (Brumfield, 2014).
8. PBPK Model Qualification and Reporting Standards (PQRS) Working Group: AstraZeneca contributes to establishing best practices for PBPK modeling through this group (Shebley et al., 2018).
9. International Society of Pharmacometrics (ISoP): AstraZeneca actively participates in ISoP with many of its scientists contributing to various Special Interest Groups (SIGs), presenting at annual meetings, and holding leadership positions within the society (Pichardo-Almarza et al., 2024; Moore, 2019).
10. American Society for Clinical Pharmacology and Therapeutics (ASCPT): AstraZeneca is actively involved in ASCPT, particularly in the Quantitative Pharmacology Network and the Model-Informed Drug Development (MIDD) Community. AstraZeneca scientists regularly contribute to ASCPT conferences, workshops, and publications, sharing insights and best practices in quantitative pharmacology and modeling approaches (Burton et al., 2023).

### 4.2 Educational and career development programs

**FIGURE 2:**
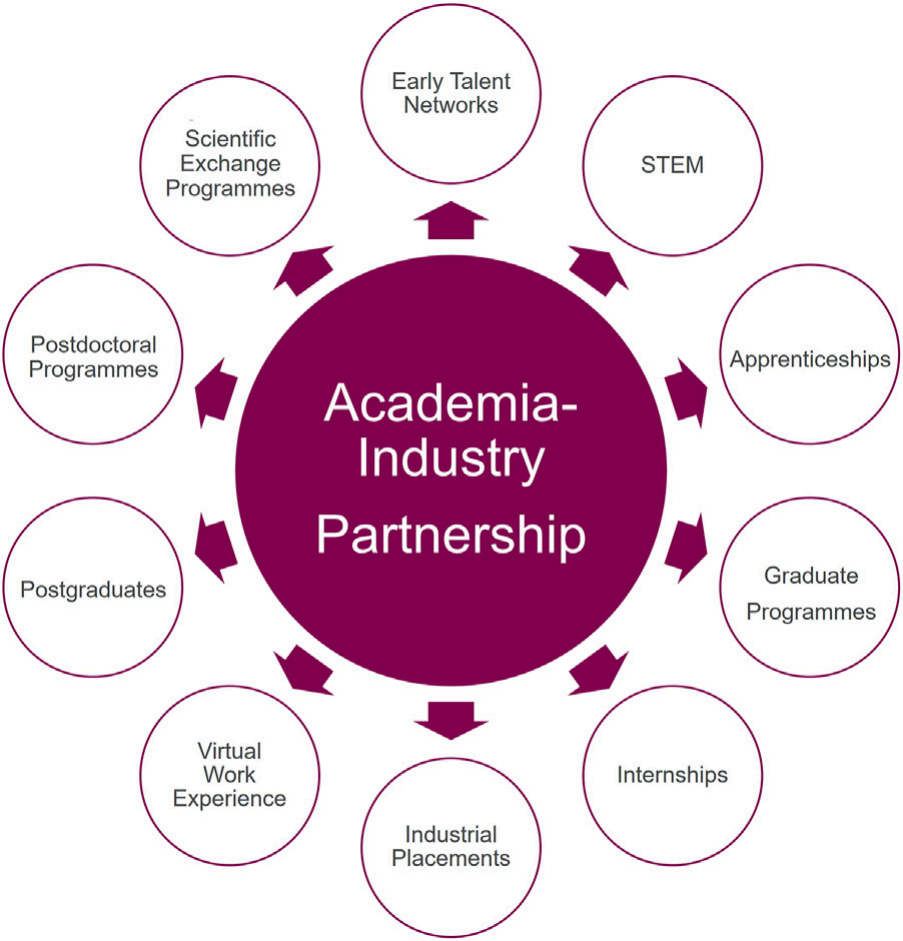
AstraZeneca educational and development programs.

AstraZeneca Educational and Development Programs (Figure 2) are summarised below with additional details shown in Table 1.

1. Graduate Programme: Offers recent graduates hands-on experience in drug discovery and development across various disciplines, including computational sciences and quantitative pharmacology.
2. Internships and Summer Research Experience: Provides opportunities for undergraduate and graduate students to work on real-world projects alongside experienced scientists.
3. Postdoctoral Programme: Allows early-career scientists to conduct cutting-edge research in an industrial setting while maintaining academic links. Any AstraZeneca scientists can submit grant proposals to compete for company-wide postdoctoral program funding. This program requires academic collaborators.
4. Pharmaceutical Industry Year Placements: Offers year-long industrial placements for undergraduate students, providing in-depth exposure to the pharmaceutical industry.
5. Collaborative Ph.D. and MSc Programs: Partners with universities for joint doctoral and master’s research projects.
6. Data Science Apprenticeship: Combines on-the-job training with academic study in data science.
7. STEM Outreach Programs: Engages younger students through various initiatives to inspire the next-generation of scientists.
8. Virtual Work Experience Programs: Provides online opportunities for students to gain insights into the pharmaceutical industry.
9. Early Talent Network: Connects students and recent graduates with resources and networking opportunities.
10. Scientific Exchange Programme: AstraZeneca participates in researcher exchanges with academic institutions, including opportunities for advanced graduate students (MTPConnect, 2022; SINAPSE, 2024).

**TABLE 1:** AstraZeneca Educational and Development Programs: additional information.

AstraZeneca programme	More info at
Early Talent Network	https://careers.astrazeneca.com/early-talent
STEM	https://www.astrazeneca.com/r-d/our-approach/stem-at-astrazeneca.html
Apprenticeships	https://careers.astrazeneca.com/digital-and-technology-apprenticeships
Graduate Programme	https://careers.astrazeneca.com/r-and-d-graduate-programme
Internships	https://careers.astrazeneca.com/early-careers-internships
Industrial Placements	https://careers.astrazeneca.com/astrazeneca-industrial-placements
Virtual Work Experience	https://www.astrazenecaworkexperience.com/welcome
Postgraduates	https://careers.astrazeneca.com/early-talent
Postdoctoral Programme	https://careers.astrazeneca.com/postdocs-astrazeneca

## 5 Recommendations for optimizing collaborations

Pharmaceutical industry and academic institutions can optimize their collaborations for productivity, sustainability, and mutually benefit by addressing the challenges and considerations discussed in Section III, particularly by developing the following areas..

Effective Leadership and Communication: Dedicated liaisons embedded in both sectors facilitate ongoing negotiation and joint problem-solving. Regular joint workshops, feedback mechanisms, and leadership networks support collaboration.

Integrated Resource Sharing and Open Science: Resource, data sharing, and IP agreements should be negotiated up-front, promoting open science where possible while protecting sensitive information.

Mentorship and Early Outreach: Structured mentorship, strongly coordinated with university careers and academic advisory services, is best for students’ growth. Industry support for challenge events, hackathons, and STEM outreach (pre-university) should be expanded.

Continuous Evaluation: Joint tracking of graduate outcomes, skills assessments, research outputs, and career progression is needed. Societies and funders are urged to support evaluation frameworks for collaborative education schemes.

## 6 Conclusion

The development of novel and effective therapies relies heavily on the expertise of well-trained scientists. These individuals should possess the unique blend of biological, mathematical, and computational skills necessary to develop and analyse complex models of biological systems, predict drug responses, and translate scientific knowledge into actionable insights. By embracing SB and QSP approaches, the pharmaceutical industry can accelerate drug development duration, reduce development costs, and ultimately bring safer and more effective therapies to patients. Cultivating a robust pipeline of skilled professionals in these areas is therefore paramount for the future of drug development.

Collaboration between academia and industry is crucial for developing the next-generation of scientific talent, addressing skill gaps, modernizing educational programs, and maintaining the practical relevance of advanced training. These partnerships create valuable opportunities for students to gain practical experience, engage with the latest technologies, and tackle real-world research problems. By working together through joint research efforts, co-created curricula, and mentorship initiatives, academic institutions and industry partners can prepare a highly skilled workforce ready to meet the evolving demands of drug development.

Looking ahead, the future of SB and QSP education demands continued innovation and collaboration. As the complexity of biological systems and the sophistication of drug development technologies continue to increase, the need for highly skilled and interdisciplinary scientists will only grow. By embracing new technologies, such as artificial intelligence and machine learning, and fostering strong collaborations between academia, industry, and other stakeholders, we can ensure that the next-generation of scientists is equipped with the knowledge and skills necessary to unlock the full potential of systems biology and QSP in advancing human health.
